# Case report: The MSI-L/p-MMR metastatic rectal cancer patient who failed systemic therapy responds to anti-PD-1 immunotherapy after stereotactic body radiation-therapy

**DOI:** 10.3389/fimmu.2022.981527

**Published:** 2022-09-02

**Authors:** Shijin Liu, Yiran Zhang, Yujian Lin, Peize Wang, Yunlong Pan

**Affiliations:** ^1^ Department of General Surgery, The First Affiliated Hospital of Jinan University, Guangzhou, China; ^2^ Ministry of Education (MOE) Key Laboratory of Tumor Molecular Biology and Key Laboratory of Functional Protein Research of Guangdong Higher Education Institutes, Institute of Life and Health Engineering, Jinan University, Guangzhou, China

**Keywords:** advanced rectal cancer, liver metastasis, immunotherapy, MSS/MSI-L/p-MMR, SBRT

## Abstract

**Background:**

Traditionally, patients with microsatellite stability (MSS)/microsatellite instability-Low (MSI-L)/proficient mismatch repair (p-MMR) metastatic colorectal cancer (mCRC) have had poor benefit from immunotherapy. Therefore, how to enhance the response of immunotherapy is still a challenge for MSS/MSI-L/p-MMR CRC patient.

**Case presentation:**

We report a special case of a rectal cancer patient with programmed death-ligand 1 (PD-L1) negative expression, MSI-L/p-MMR, tumor mutational burden-low (TMB-L) and liver metastases, who partial response (PR) to immunotherapy after systemic therapy failure including chemotherapy, anti-angiogenesis therapy and stereotactic body radiation-therapy (SBRT). The computed tomography (CT) results showed that among three liver metastases had been reduction or disappearance after Tislelizumab treatment for three times. Besides, the carcinoembryonic antigen (CEA) and carbohydrate antigen 199 (CA199) decrease and maintained at a low level for 3 months. The progression-free survival (PFS) of patient has exceeded 3 months.

**Conclusions:**

This case indicates that the patient with MSI-L/p-MMR mCRC can respond to anti-PD-1 immunotherapy after systemic therapy. And the SBRT (targeting liver metastases) may a method for increase-sensitivity of immunotherapy in CRC patients with MSI-L/p-MMR.

## Case presentation

**Figure 1:**
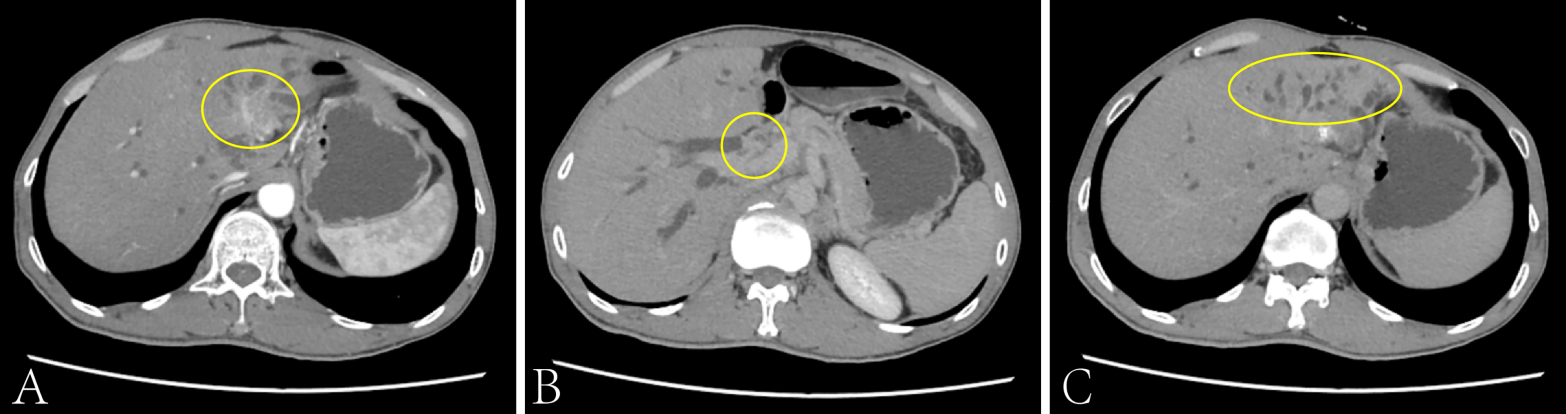
The CT scan of the patient’s liver on Dec. 2020. The main metastatic lesion (3.4 × 2.6cm) in the left and caudate lobe of the liver **(A)**, the metastatic nodule (1.5 × 1.1cm) in the hepatic portal **(B)** and the localized common bile duct compression **(C)**.

A 49 years old man was admitted to our hospital on December 21, 2020 with the rectal cancer liver metastasis for more than 1 year. The CT results showed that a main metastatic lesion (3.4 × 2.6 cm) in the left and caudate lobe of the liver, a metastatic nodule (1.5 × 1.1 cm) in the hepatic portal and the localized common bile duct compression (**Figure 1**). The puncture pathology biopsy of liver metastases shows moderately differentiated tubular adenocarcinoma of colorectal origin. And the next-generation sequencing (NGS) result showed that the tumor with MSI-L (11.11%), PD-L1 negative expression and TMB-L (8.2 Muts/Mb). The serum biomarker showed CEA and CA199 at 17.68 ng/ml and 1109.89 u/ml, respectively, and normal levels of alpha-fetoprotein (AFP). The patient received a radical surgery when rectal cancer initial diagnosis in February 2018. The TNM stage was T2N0M0. Follow-up observations found that tumor liver metastases in July 2019, then, he has received six cycles of conversion therapy (CapeOX + Bevacizumab) and nine cycles of maintenance therapy (Capecitabine + Bevacizumab) until disease progression on December 21, 2020.

**Figure 2:**
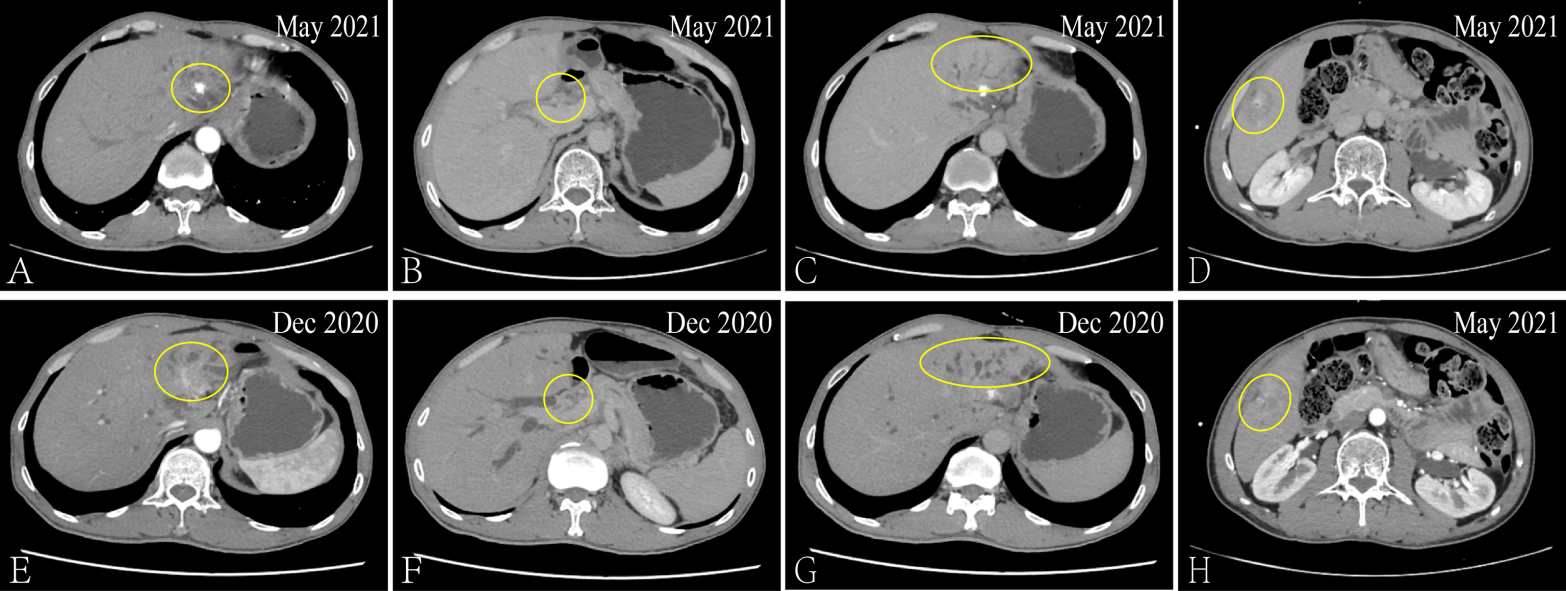
*Via* CT to contrast the curative effect between the May 2021 and Dec 2020. The size of the metastatic lesion which in the left and caudate lobe of liver is reduction from 3.4 × 2.6cm to 2.6 × 2.1cm **(A, E)**. The hepatic portal metastatic nodule has disappeared **(B, F)** and the compressive bile duct has improvement **(C, G)**. The new metastatic lesion (2.7 × 1.7cm) in liver S5 **(D, H)**.

**Figure 3:**
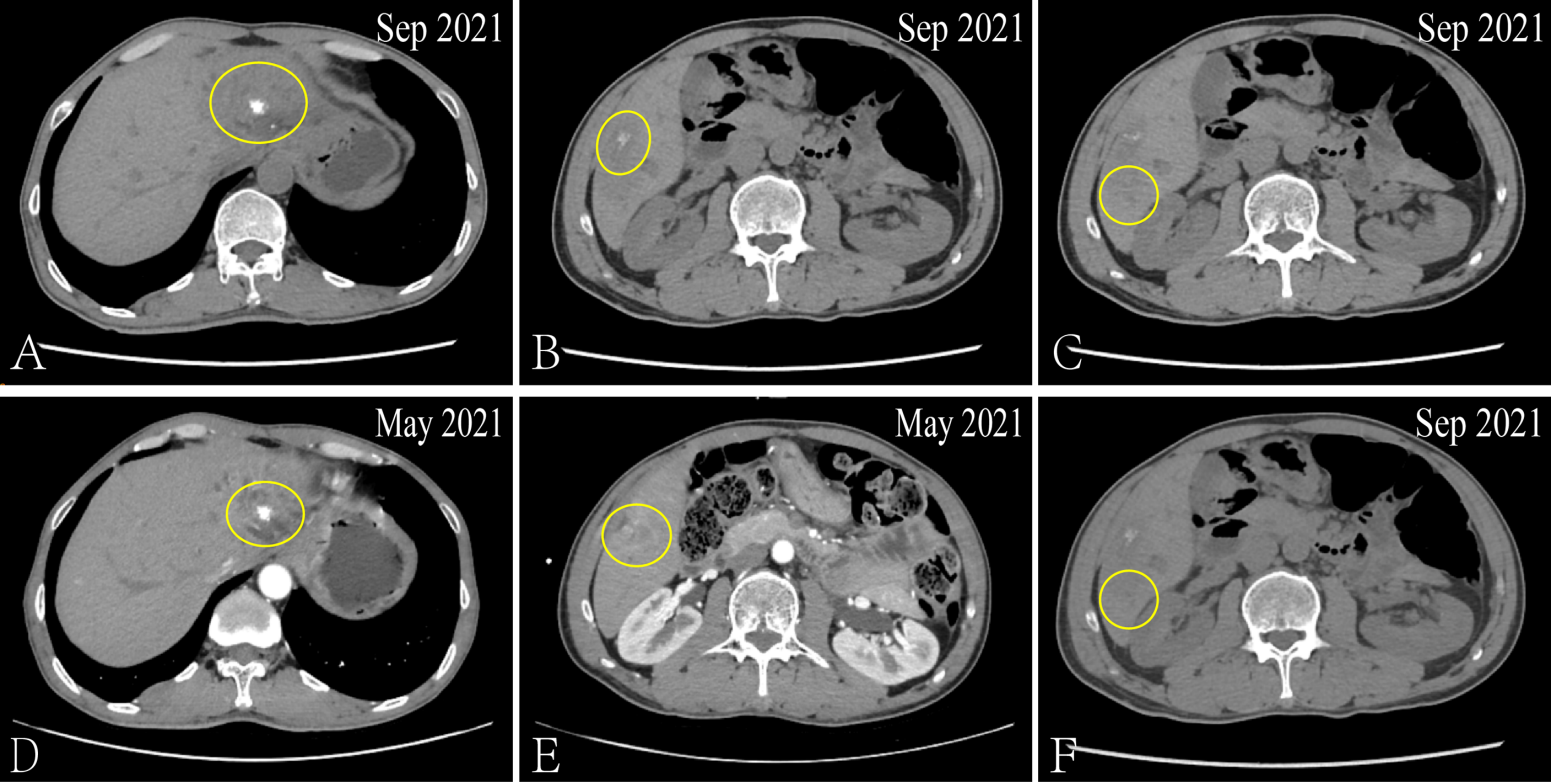
*Via* CT to contrast the curative effect between the Sep 2021 and May 2021. The size of the metastatic lesion which in the left and caudate lobe of liver is increase from 2.6 × 2.1cm to 3.3 × 2.6cm **(A, D)**. The size of the metastatic lesion which in liver S5 is reduction from 2.7 × 1.7cm to 2.3 × 1.1cm **(B, E)**. The new metastatic lesion (1.5 × 1.2cm) in liver S6 **(C, F)**.

After admission, the patient received the FOLFIRI combined with Cetuximab treatment, And each liver metastatic lesions received SBRT, the radiotherapy dose was 350 cGy/d with ten times. From December 21, 2020 to May 27, 2021, the serum biomarker showed a decrease in CEA and CA199 to 6.02 ng/ml and 85.54 u/ml, respectively. And the CT results showed that the metastatic lesion which in the left and caudate lobe of liver is reduction, the hepatic portal nodule has disappeared and the compressive bile duct has improvement. However, there’s a new metastatic lesion (2.7 × 1.7cm) in liver S5 (**Figure 2**). Thus, the curative effect was evaluated as progressive disease (PD) with a PFS of 5 months. So, the SBRT was aimed at the new liver’s lesion (S5) again in May 31, 2021, and the Regorafenib (third-line therapy) is added for treatment. Similarly, the CT results showed a new metastatic lesion (1.5 × 1.2cm) in liver S6 on September 03, 2021 (**Figure 3**). And the serum biomarker showed elevated CA199 to 11613.09 u/ml, with CEA and AFP at normal levels. Therefore, the curative effect was assessed as PD with a PFS of 3 months.

**Figure 4:**
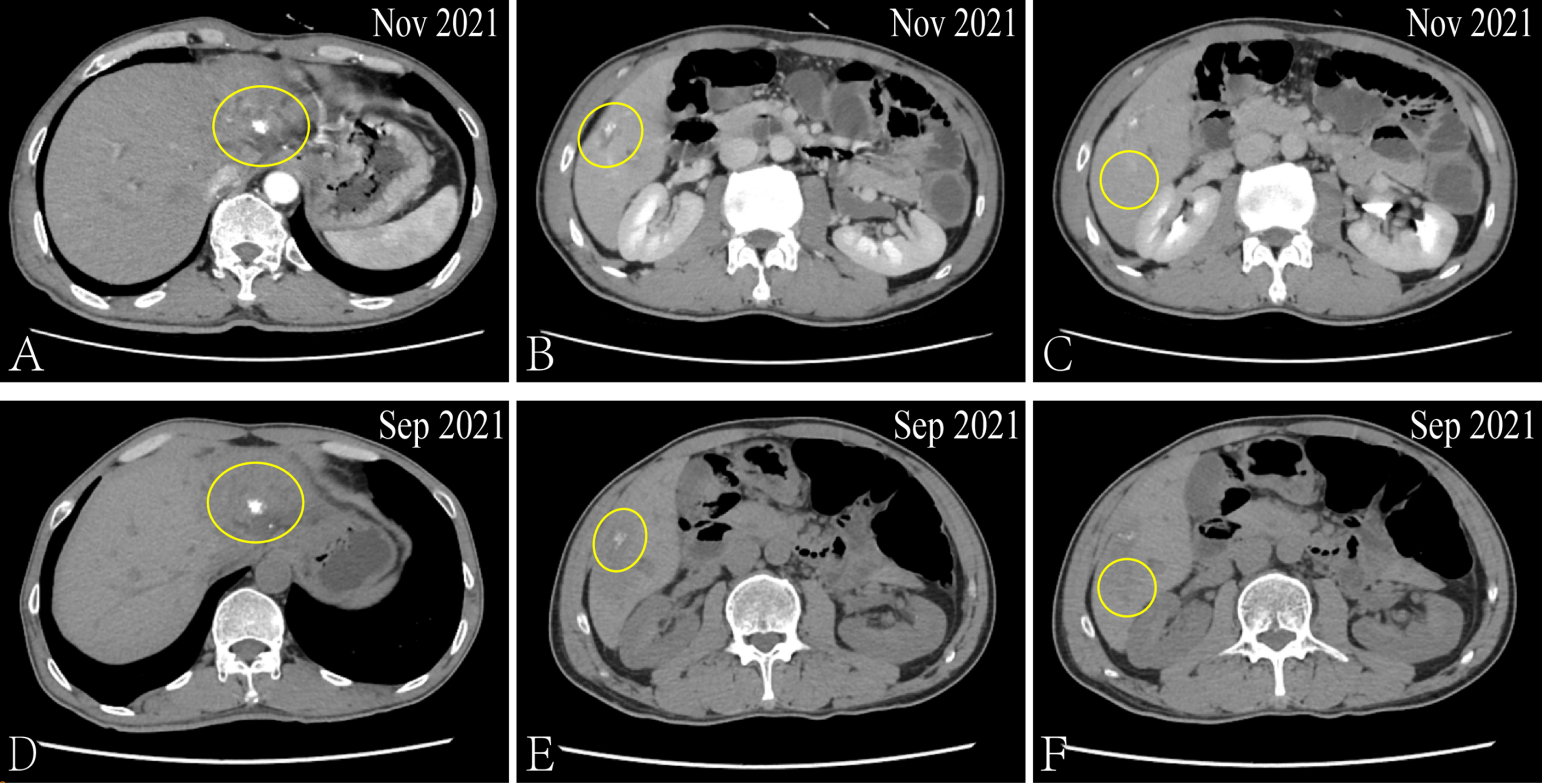
*Via* CT to contrast the curative effect between the Nov 2021 and Sep 2021. The size of the metastatic lesion which in the left and caudate lobe of liver is reduction from 3.3 × 2.6cm to 2.8 × 2.2cm **(A, D)**. The size of the metastatic lesion which in liver S5 is reduction from 2.3 × 1.1cm to 2.0 × 0.9cm **(B, E)**. The metastatic lesion in liver S6 has disappeared **(C, F)**.

**Figure 5:**
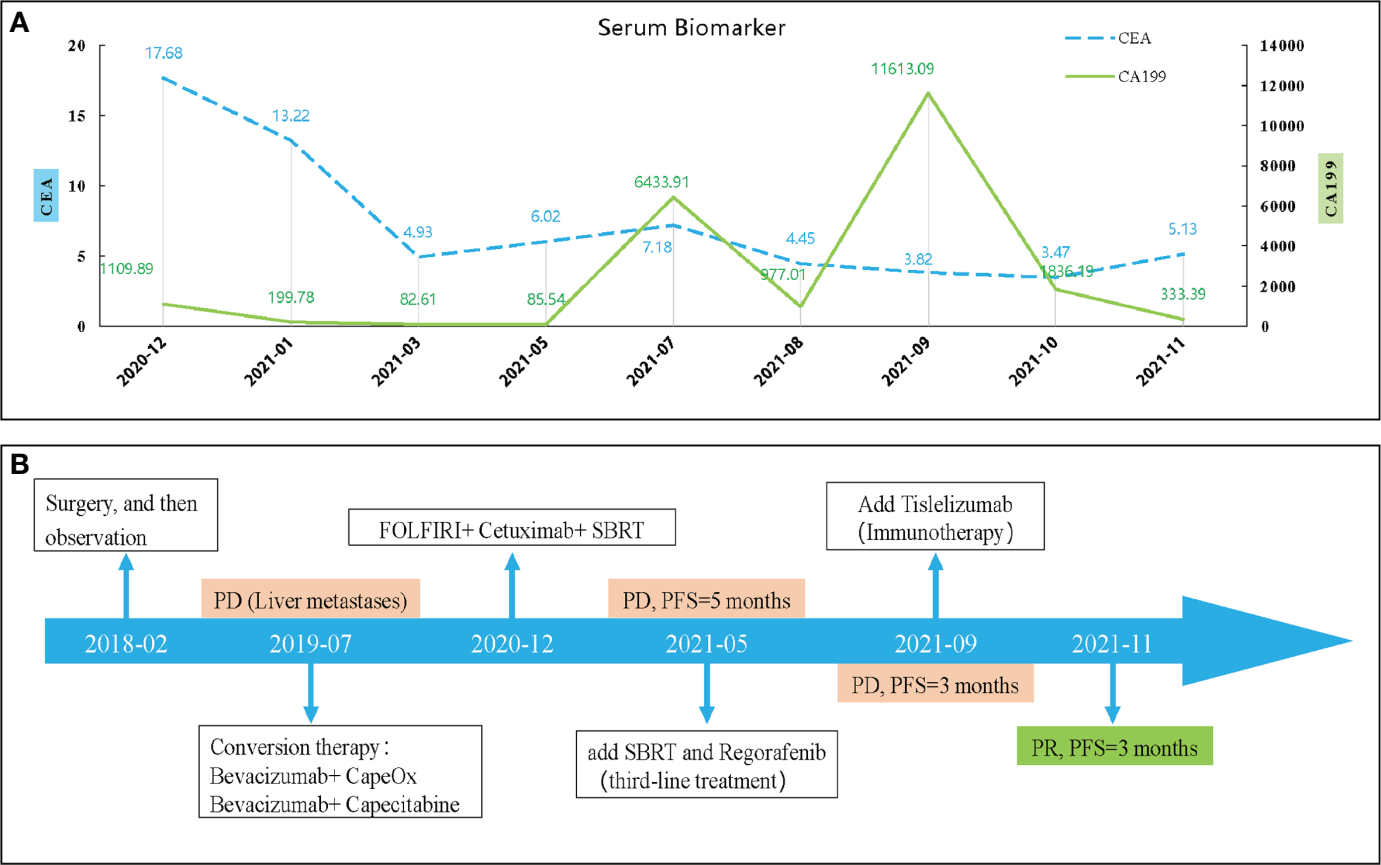
The change of serum biomarker **(A)** and timeline of treatment **(B)**. CEA, carcinoembryonic antigen; CA199, carbohydrate antigen 199; PD, disease progression; SBRT, stereotactic body radiation-therapy; PFS, progression-free survival.

So far, the disease continued to progress rapidly after using first-, second- and third-line treatment. The patient was decided to receive the first dose of anti-PD⁃1 drug (Tislelizumab) on September 04, 2021 after obtaining the consent of the patient and his family after. Surprisingly, after three cycles, the CT results showed that the metastatic lesion which in the left and caudate lobe and S5 of liver is reduction and the lesion of S6 has disappeared (**Figure 4**). And the serum biomarker showed a significantly decrease in CA199 from 11613.09 u/ml to 333.39 u/ml. Therefore, the curative effect was assessed as PR with a PFS of 3 months after the anti-PD⁃1 therapy. The change of serum biomarker and timeline of treatment was shown in **Figure 5**.

## Discussion

Worldwide, the CRC is one of the most common cancers and the third leading cause of cancer-related deaths (1). Especially, for mCRC patients, the 3-year survival rate is only about 30%, while the survival of MSS/MSI-L/p-MMR patients is even lower (2). The current primary treatment for unresectable mCRC is systemic therapy (e.g., chemotherapy, radiation therapy, targeted therapy, immunotherapy, and combinations of them) (3). Today, immunotherapy has shown significant efficacy in a variety of solid tumors, but appears to benefit only 5% of mCRC patients (those with high microsatellite instability (MSI-H)/defective mismatch repair (d-MMR)) (4). Therefore, the application of immune-therapy in mCRC patients (especially with MSS/MSI-L/p-MMR patients) remains full of challenges.

Generally, the PD-L1 positivity expression, MSI-H/d-MMR and tumor mutational burden-high (TMB-H) are considered as predictors of effective immunotherapy (5). However, it has been shown that the expression of PD-L1 is not the same in primary and metastatic sites of colorectal cancer, and even the PD-L1 status does not correlate with both PFS and Overall survival (OS) in some MSS/p-MMR CRC patients (6, 7). And the predictive effects of TMB or MSI status may not be applicable to all solid tumors (8). In addition, the status of PD-L1, TMB and MSI/MMR may not be reliable predictors due to assay methods, different cut-off value settings, etc. Interestingly, the patient is an exception to the classical prediction: PD-L1 negative expression, MSI-L/p-MMR and TMB-L, but with a partial response (PR) to immunotherapy. Therefore, in order to benefit more patients with MSS/MSI-L/p-MMR CRC from immunotherapy, it is necessary to find the reasons for the failure of classical predictors in this case and to reveal the possible factors.

More and more studies are exploring how to transform the “cold” tumor of MSS into the “hot” tumor of MSI-H. One of the most important strategies is radiation therapy (RT) combined with immunotherapy. Although, the radiofrequency ablation (RFA) is considered to be the first treatment for unresectable liver metastases, RFA has limitations for some lesions, such as its size larger than 3 cm and adjacent to important vessels or bile ducts (9). Besides, some studies has been shown that there is no significant difference in OS between the SBRT or RFA treatment for liver metastases. But, for the size larger than 2 cm tumors, SBRT improves the freedom from local progression (FFLP) more than RAF (10). Thus, as one of the important treatments for liver metastases in CRC patients, RT (especially SBRT), may have a better prospect. It has been shown that it can trigger type I IFN response and activate anti-tumor T cells, through cGAS-STING signaling pathway, and improve tumor immune microenvironment, thus synergistically enhancing anti-tumor effects (11, 12). RT is also an effective “immune booster”, and the immunotherapy tolerance in multiple progressive MSS/p-MMR CRC patients with liver metastases can be overcome even by the local immunomodulatory therapy such as SBRT (13, 14). In addition, Satoshi et al. showed that chemoradiotherapy sequenced with nivolumab was effective in treating patients with locally advanced rectal cancer with MSS (15). The study (NCT02437071) by Segal et al (16) also indicated an objective response in non-irradiated lesions after the application of RT combined with pembrolizumab in MSS/p-MMR CRC patients (although the ORR was only 9%). Likewise, regorafenib and others have similar effects. For example, a study (NCT03406871) showed a significant effect of regorafenib in combination with nivolumab in 24 MSS/p-MMR advanced CRC patients and the overall objective response rate (ORR) reached 33%, but only two patients with liver metastases (2/13) (17) Fakih et al (18)demonstrated that regorafenib combined with nivolumab achieved an ORR of 7.1% in 70 MSS mCRC patients. However, the result also showed that none of the liver metastases patients (0/47). Overall, some MSS/p-MMR advanced CRC patients are effective for combination immunotherapy. Although the exact mechanism is still unclear, based on the current findings, RT combined with immunotherapy seems to better promote the benefit of immunotherapy in MSS/p-MMR CRC patients with liver metastases. So, this may explain why the patient (with PD-L1 negative, MSS/p-MMR, TMB-L and liver metastases only) responded well to immunotherapy after systemic treatment failure. For our case, one of the most important factors may be SBRT, but this also needs and deserves more relevant studies to verify.

## Conclusion

We report a novel case of PD-L1 negative expression, MSI-L/p-MMR, TMB-L and with liver metastases rectal cancer patient who obtained PR and the PFS has exceeded 3 months after immunotherapy. This case indicates that the patient with MSI-L/p-MMR mCRC can respond to anti-PD-1 immunotherapy after systemic therapy. And the SBRT (targeting liver metastases) may a method for increase-sensitivity of immunotherapy in CRC patients with MSI-L/p-MMR.

## Data availability statement

The original contributions presented in the study are included in the article. Further inquiries can be directed to the corresponding author.

## Ethics statement

Written informed consent was obtained from the individual(s) for the publication of any potentially identifiable images or data included in this article.

## Author contributions

SL and YZ were mainly responsible for the article writing. YL and PW were responsible for patient’s clinical data and analysis. YP was the corresponding author. All authors contributed to the article and approved the submitted version.

## Funding

This research was supported by the Clinical Frontier Technology Program of the First Affiliated Hospital of Jinan University, China (No. JNU1AF-CFTP-2022-a01223), Natural Science Foundation of Guangdong Province (2019A1515011763; 2020A1515110639; 2021A1515010994; 2022A1515011695), Guangzhou Science and Technology Plan City-School Joint Funding Project (202201020084; 202201020065), the Fundamental Research Business Expenses of Central Universities (21620306).

## Conflict of interest

The authors declare that the research was conducted in the absence of any commercial or financial relationships that could be construed as a potential conflict of interest.

## Publisher’s note

All claims expressed in this article are solely those of the authors and do not necessarily represent those of their affiliated organizations, or those of the publisher, the editors and the reviewers. Any product that may be evaluated in this article, or claim that may be made by its manufacturer, is not guaranteed or endorsed by the publisher.
